# Evaluation of ergonomic and education interventions to reduce occupational sitting in office-based university workers: study protocol for a randomized controlled trial

**DOI:** 10.1186/1745-6215-14-330

**Published:** 2013-10-12

**Authors:** Antonia Radas, Martin Mackey, Andrew Leaver, Anna-Louise Bouvier, Josephine Y Chau, Debra Shirley, Adrian Bauman

**Affiliations:** 1 Faculty of Health Science, The University of Sydney, 75 East Street, Lidcombe, Sydney, NSW 2141, Australia; 2 Physiocise Movement for Muscles Pty Ltd, Suite 14, 77 Penshurst Street, Willoughby, NSW 2068, Australia; 3 Prevention Research Collaboration, Sydney School of Public Health, The University of Sydney, K25 - Medical Foundation Building The University of Sydney, Sydney, NSW 2006, Australia

**Keywords:** Sedentary behavior, Musculoskeletal symptoms, Ergonomic, Work posture, Intervention, Behavior change

## Abstract

**Background:**

Prolonged sitting is a specific occupational hazard in office workers. There is growing evidence that prolonged sitting is detrimental to metabolic health. The aim of this study is to determine whether providing office workers with education along with adjustable sit-stand workstations leads to reduction in sitting behavior.

**Methods/Design:**

A randomized control trial (RCT) with three groups (one control group and two intervention groups) will be conducted in an office workplace setting. The education intervention group will receive an education package that encourages reduction in sitting behaviors. The sit-stand desk intervention group will receive the same education package along with an adjustable sit-stand desk. Participants will be included in the study if they are currently employed in a full-time academic or administrative role that involves greater than 15 hours per week or greater than 4 hours per day computer-based work. Baseline data will include participant’s age, gender, weight, height, smoking habit, employment position, level of education, and baseline self-reported leisure time physical activity. The primary outcome is the average daily sedentary time during work hours, measured by an accelerometer. Participant recruitment commenced in March 2013 and will be completed by December 2013.

**Discussion:**

This study will determine whether providing office workers with an adjustable sit-stand desk and individually targeted education, or education alone, is more effective in decreasing sitting behaviors than no intervention.

**Trial registration:**

Australian New Zealand Clinical Trials Registry: ACTRN12613000366752

## Background

One of the features of modern working life is that jobs are becoming increasingly less active and more sedentary [1]. Prolonged sitting has been identified as a potentially significant occupational health concern [2], as higher levels of occupational sitting have been linked to increased prevalence of chronic diseases including coronary heart disease [3], diabetes [4], obesity [5], and breast cancer [6], as well as increased mortality from all causes [7]. Risk of musculoskeletal disorders has also been linked to increased exposure to sitting at work [8]. Given that average working hours have generally increased over the past 32 years [9], with adults now spending an average of more than 8 hours of their weekday at work [10], the workplace is a key setting in which to introduce strategies to reduce sitting time and break up periods of prolonged sitting to improve health [11,12]. Prolonged sitting is of particular concern in certain occupational groups such as office workers [13]. High rates of sedentary behaviors have been demonstrated in particular groups of office workers including managers, professionals, clerical and administrative workers [14], public service workers [8], sales people [15], and health company workers [16]. One reason for this might be the shift to the 'paperless office’ and the growing prevalence of computerized work environments [1]. Observational studies of Australian office workers have demonstrated that up to two-thirds of a working day, or half of an office-worker’s waking hours are spent in sedentary postures [13,17].

Both education and ergonomic interventions have been trialed in the office workplace environment in an attempt to increase incidental physical activity and reduce sedentary behavior [18]. Education interventions typically employ behavior change strategies such as goal-setting, self-monitoring, and use of external cues [19,20]. There is some evidence that strategies such as using pedometers, reducing the use of seated telephone and email time in favor of face to face contact, using a bathroom further away from the office workstation, and having standing and/or walking meetings can increase incidental physical activity at work [21]. Several studies that have evaluated the effect of education interventions have used an approach based on the transtheoretical (stages of change) model [22]. This approach matches the education strategy to the individual participant’s readiness to embrace behavioral change.

A number of ergonomic interventions have also been investigated as a means of reducing unhealthy sitting behavior or increasing energy expenditure in office workers. These include walking workstations [23,24], portal pedal machines [25], and the use of adjustable sit-stand workstations [11,26]. Of these, adjustable sit-stand desks [11,26] show particular promise. The sit-stand desk is an adjustable workstation, which attaches to the worker’s desk allowing adjustment of the height of the computer monitor and keyboard allowing work in a sitting or standing position. This device allows the worker to quickly and easily change working position from sitting to standing enabling workers who are seated for long periods of their workday to have frequent standing breaks. There is some evidence that sit-stand desks are effective. A quasi-experimental study [11] demonstrated reduction in sitting time between 61 and 137 minutes/day when using one of these devices. However, these results have not been replicated in a randomized controlled trial (RCT). To date there has been no investigation of the effect of targeted education (incorporating behavior change) alone, compared with education plus access to an ergonomic device such as an adjustable sit-stand workstation, on reducing sedentary behavior in office-based workers. Although preliminary studies investigating these interventions applied separately have demonstrated promising results [11,21,23-26], no single intervention appears to suit all workers. It is also possible that education approaches and ergonomic interventions might have complementary effects.

The aim of this study is to determine whether providing office workers an adjustable sit-stand workstation together with targeted education, or targeted education alone leads to changes in sedentary behavior at work compared with no intervention. A secondary aim is to determine if one intervention is more effective than the other in affecting sedentary time.

## Methods

### Trial design

**Figure 1:**
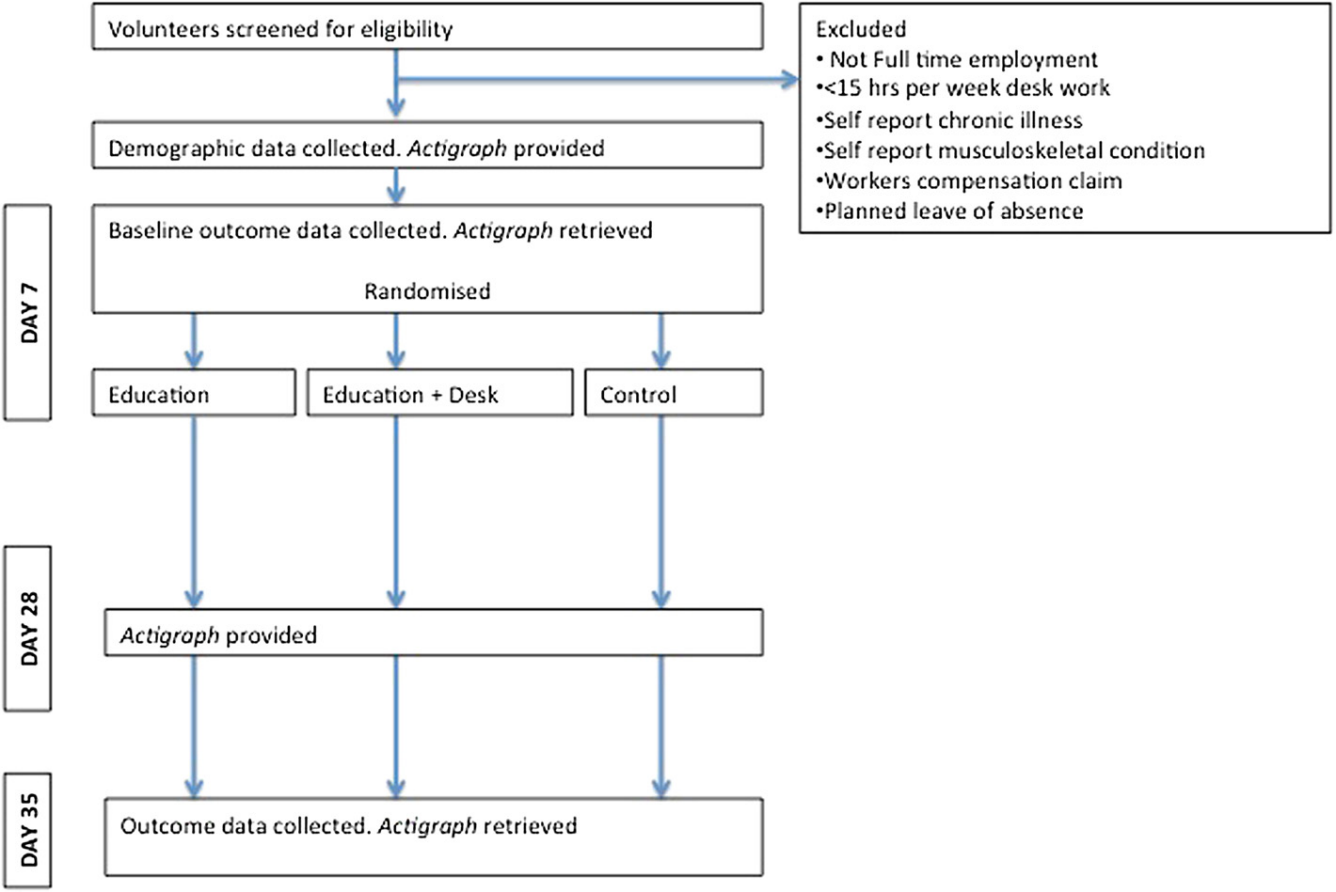
Flow of participants through trial.

A RCT with two intervention groups and one control group will be conducted in an office workplace setting. Participants will be recruited from academic and administrative staff of The University of Sydney, Sydney, Australia. The study has been approved by The University of Sydney Human Research Ethics Committee, protocol number 15448. Written informed consent will be obtained from each participant prior to entry into the trial. The trial is registered with the Australian New Zealand Clinical Trials Registry, ACTRN12613000366752. The flow of participants through the trial is demonstrated in Figure 1.

### Participants

Sixty participants will be recruited by advertisement from academic and administrative staff at the Faculty of Health Sciences, The University of Sydney. Posters will be placed in staff tearooms and common areas, inviting staff to participate in 'The Healthier Office Study’. The advertisements will contain general information informing participants that we are testing simple occupational health interventions and that participants will be provided with an ergonomic device or advice about improving healthy work practices. The advertisements will not contain specific detail about the interventions in order to keep the participants blinded to the interventions that they do not receive. The study will also be advertised in a short presentation at Faculty staff meetings to improve potential participants’ awareness of the study.

Participants will be included in the study if they are currently employed in a full-time academic or administrative role that involves greater than 15 hours per week or greater than 4 hours per day of computer-based work. The number of hours spent performing desk or computer duties will be determined for the purpose of inclusion in the study by self-report. Participants will be excluded if they have planned leave during the study period, any self-reported chronic illness, any self-reported musculoskeletal condition, or if they have a current worker’s compensation claim.

Data collection will involve the participant completing standardized assessment forms developed for this study. The assessment forms were piloted prior to commencement of the study to check for readability and participant burden. It was established that data collection for each participant involved less than 10 minutes of participant’s time. Missing data will be collected by telephone or email contact with the participant.

### Interventions

Participants will be randomly allocated to one of three study groups.

In the education intervention group, participants will receive an education package based on the 'Happy Body at Work’ program developed by a health promotion expert and physiotherapist (ALB). The education package was specifically designed for the participants in this study in consultation with the program author. It incorporates evidence-based principles of optimal seated posture, advice concerning regular postural change between sitting and standing, and physical activity guidelines for promoting a healthy lifestyle. The education package will be delivered by a researcher (ANR) who has received training in health promotion and program content from ALB. The aims of the education package are to improve the participants’ knowledge and understanding about the detrimental effects of prolonged sitting, and to motivate and engage the participants in behavior change in relation to sitting habits. The contents of the program include a multimedia presentation, a physical activity goal-setting exercise, self-monitoring of breaks from sitting, and self-monitoring of daily step count, as well as visual and auditory reminders about taking breaks from sitting.

In the sit-stand desk intervention group, participants will be provided with a WorkFit-S or WorkFit-A adjustable sit-stand workstation (Ergotron, Saint Paul, MN, USA) for a period of 4 weeks. Participants will receive the same education package as the education intervention group. Participants will also receive training in the use of the workstation by a member of the research team. Participants will set individual goals for the time that they will spend working in a standing position. Participants will be given the general advice to alternate regularly between work in a sitting and standing position, and discouraged from trying to only work in a standing position.

In the control (no intervention) group, participants will receive no information or advice about postural change and no modification to their office desk set-up. Participants in the control group will be offered the advice intervention once they have completed final data collection and they have been discharged from their involvement in the study.

## Trial status

At the date of manuscript submission participant recruitment had commenced but had not been completed.

### Outcome measurements

#### Baseline measurements

A researcher who is blinded to subject allocation will record baseline and outcome data. Baseline data will be collected from participants at the initial assessment to obtain a profile of the participant’s demographic, work, and general health characteristics. This profile will include the participant’s age, gender, weight, height, smoking habit, employment position, and level of education. Baseline self-reported leisure time physical activity will also be measured at this point using the Active Australia Questionnaire [27]. Recall instruments of physical activity such as the Active Australia Questionnaire correlate with actual physical activity measures such as accelerometer data [28].

#### Primary outcomes

The primary outcomes are the average daily sedentary time during work hours and the average number of breaks per day. These will be measured over a 7-day period immediately prior to randomization and at the completion of the intervention period. Sedentary time will be measured using an accelerometer (ActiGraph GT3XP, ActiGraph, Pensacola, FL, USA) and participants will be required to keep a log of work hours during these measurement periods. Following this period all data will be downloaded and analyzed. The ActiGraph accelerometer is a small, lightweight, plastic device worn around the waist. It measures motion data using three axes. This device is reliable and provides stable measurements of physical activity when compared to other measures of physical activity [29].

#### Secondary outcomes

The secondary outcomes include self-reported sitting time, musculoskeletal symptoms, and workability. Self-reported sitting time will be established using the Occupational Sitting and Physical Activity Questionnaire [30] and the Workforce Sitting Questionnaire [31]. Both the Occupational Sitting and Physical Activity Questionnaire and Workforce Sitting Questionnaire are acceptable self-report measures for assessment of sitting time at work. The Occupational Sitting and Physical Activity Questionnaire has high test-retest reliability [30], and both questionnaires correlate well with accelerometry measures of physical activity and sedentary time making them suitable tools for this study [30,31]. The 7-day prevalence of musculoskeletal symptoms will be recorded using the Nordic Musculoskeletal Questionnaire [32]. Self-reported work ability will be measured by the Work Ability Index [33]. These instruments have acceptable reliability and validity [34-36], and measures will be recorded at baseline and 4-week follow-up.

Potential adverse effects of the interventions will be recorded at 4-week follow-up. Participants will be asked to recall if they experienced any adverse effects that they related to their involvement in the study from a list that includes: neck pain, headache, back pain, other muscle or joint pain, fatigue, loss of concentration, and work productivity.

#### Sample size calculations

We did not conduct a formal power analysis to determine sample size, since the threshold for defining harmful levels of sitting is unknown. The proposed sample size of this study is similar to previous pilot studies of workplace sitting and sedentary time. Our sample of 60 participants (three groups of 20 participants) should be sufficient for assessing the direction of intervention effects, while taking into account possible missing data and participation attrition.

#### Randomization and blinding

Participants will be randomly allocated into one of three study groups. A researcher who is not involved in participant recruitment or data collection will produce a randomly generated sequence for allocating participants to one of the three groups. This will be done prior to commencement of the trial. This researcher will prepare consecutively numbered sealed opaque envelopes that contain the group allocation for each individual participant. The envelope for each participant will be opened after the participant has enrolled in the study and after baseline data is collected. The randomization sequence will contain equal numbers of subjects in each group but will be otherwise unrestricted.

Data collection will take place away from the participants’ office and data collectors will be instructed not to enter the office areas of participants. This will ensure blinding of the data collectors to allocation of participants to the sit-stand desk intervention group. The office set-up in the workplace setting for this trial is predominantly private, single occupier offices with few work centers using open-plan office design. This feature of the study setting will assist with limiting the exposure of participants to the other trial interventions and ensure blinding of the data collectors to treatment allocation.

Participants will be kept naive as to the main aim of the study, that is, reduction of unhealthy sitting behaviors. This is because it is believed by the research team that participants being aware that the primary outcome is to reduce sitting behavior might actually change this behavior. Participants will also be blinded where possible to the intervention groups to which they are not allocated. Knowledge of the other interventions that are being tested, both clearly being interventions designed to reduce sitting behavior, might also impact on the sitting behavior of participants. Participants will therefore be informed that they are participating in a trial of general workplace health interventions and remain blinded to the other intervention groups.

A researcher who is blinded to the participant allocation will perform data entry and analysis.

#### Statistical methods

Accelerometer activity counts will be recorded in 1-second intervals and aggregated into 1-minute epochs. We will download ActiGraph data using ActiLife proprietary software and conduct further processing with a custom macro to categorize the data into activity intensity categories: sedentary (<100 counts/minute), light (101 to 2,020 counts/minute), moderate (2,021 to 5,999 counts/minute), and vigorous (≥6,000 counts/minute), based on the frequently used sedentary cut-point for ActiGraph accelerometers [37] and National Health and Nutrition Examination Survey (NHANES) cut-points for light, moderate, and vigorous intensity levels [38]. Spurious epochs will be defined as over 20,000 counts/minute [39], and non-wear time will be defined as periods of consecutive strings of zero-count epochs lasting at least 60 minutes. A whole day of monitoring will be considered as valid if the participant wears the accelerometer for at least 10 hours during their waking time. A workday will be considered valid if the participant wears the accelerometer for at least 75% of their time at work [40].

We will conduct two-way repeated measures analyses of variance (rANOVA) to compare participants’ objectively assessed time spent in sedentary, light, and moderate-to-vigorous intensity physical activity, as well as their self-reported sitting time, musculoskeletal symptoms, and workability pre- and post-intervention. The two-way rANOVA will have one group factor (education; education plus sit-stand desk; control) and one time factor (pre- and post-intervention). Models will test for group and time main effects and group X time interactions to examine whether any differences in outcome variables vary by study group from pre- to post-intervention.

The primary analysis will be by intention-to-treat. All analyses will be conducted using IBM SPSS Statistics for Windows Version 21.0 (IBM, Armonk, NY, USA).

## Discussion

This paper outlines the rationale and design for a RCT that compares the effectiveness of two simple occupational health interventions in reducing sitting behavior in office workers.

Our recruitment strategy introduces potential limits to the generalizability of the results of this study, which we acknowledge *a priori*. Since we will recruit participants into the study using advertisements that target people who are interested in improving their healthy work practices, the results might not generalize to all office workers. It is possible that a more intensive education strategy might be required for workers who have not contemplated changing work practices for health reasons. In these workers the education package might need to focus more on motivating a desire to change sitting behavior.

This study will determine whether providing office workers with an adjustable sit-stand desk and individually targeted education is more effective in decreasing sitting behaviors than education alone or no intervention. We will establish whether these interventions change sitting behavior by comparing participant activity levels using accelerometers and by self-report. In addition to these measures we will also investigate the impact of sitting and changes in sitting behavior on musculoskeletal symptoms and workability. This information is important, as few studies have investigated the impact of changing sitting on musculoskeletal health and work ability in an office workplace. The outcomes of this pilot study will also provide evidence to inform the further development of existing guidelines and health policy concerning workplace sitting [41,42].

## Abbreviations

NHANES: National Health and Nutrition Examination Survey; rANOVA: repeated measures analyses of variance; RCT: Randomized controlled trial.

## Competing interests

The authors declare that they have no competing interests.

## Authors’ contributions

Each author made a substantial contribution to the study protocol and/or drafting of the manuscript. AR was responsible for trial implementation and drafted the manuscript. MM conceived, contributed to the design of, and secured funding for the trial. AL was responsible for coordination of the trial and allocation sequencing. A-LB designed the education intervention. JYC participated in the design of trial and was responsible for the statistical analysis. DS participated in data collection. AB participated in the design of trial, and provided seed funding and in-kind support. All authors read and approved the final manuscript.
